# Supplementary material for: Pathogenic Vibrio Species Are Associated with Distinct Environmental Niches and Planktonic Taxa in Southern California (USA) Aquatic Microbiomes
Source: mSystems. 2021 Jul 6;6(4):e00571-21. doi: 10.1128/mSystems.00571-21 (PMC8407410; doi:10.1128/mSystems.00571-21)
Supplement: FIG S1 [file msystems.00571-21-sf001.pdf]

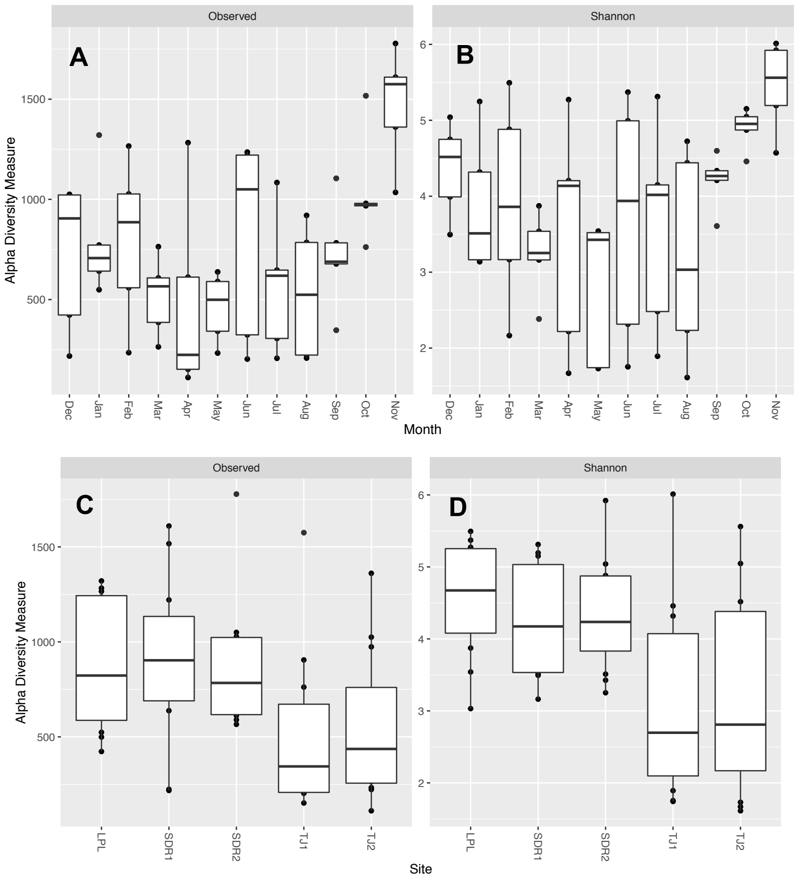

E

	Statistic (Kruskal-Wallis chi-squared)	*p-value*
Observed by Site	13.04	0.011
Observed by Month	22.25	0.023
Shannon by Site	13.35	0.010
Shannon by Month	22.80	0.019
