# Supplementary material for: Pathogenic Vibrio Species Are Associated with Distinct Environmental Niches and Planktonic Taxa in Southern California (USA) Aquatic Microbiomes
Source: mSystems. 2021 Jul 6;6(4):e00571-21. doi: 10.1128/mSystems.00571-21 (PMC8407410; doi:10.1128/mSystems.00571-21)
Supplement: FIG S2 [file msystems.00571-21-sf002.pdf]

A. Random forest classification

16S amplicon data			
**Variable**	**Accuracy ratio**	**Baseline accuracy (%)**	**Overall accuracy (%)**
Month (*n=12*)	11	8.33	91.67
Site (*n=5*)	4	20.83	83.33
18S amplicon data			
Month (*n=12*)	6	8.33	50.00
Site (*n=5*)	2.4	20.83	50.00

B. Random forest regression

	16S amplicon data				
**Variable**	**R-value**	**p-value**	**R-square**	**Mean squared error (MSE)**	**Std Error**
Temperature	0.90	1.54e-09	0.82	7.53	0.061
Salinity	0.85	1.61e-07	0.72	23.85	0.088
	18S amplicon data				
Temperature	0.47	0.019	0.22	25.55	0.085
Salinity	0.44	0.032	0.19	75.63	0.068
