# Supplementary material for: Pathogenic Vibrio Species Are Associated with Distinct Environmental Niches and Planktonic Taxa in Southern California (USA) Aquatic Microbiomes
Source: mSystems. 2021 Jul 6;6(4):e00571-21. doi: 10.1128/mSystems.00571-21 (PMC8407410; doi:10.1128/mSystems.00571-21)
Supplement: FIG S3 [file msystems.00571-21-sf003.pdf]

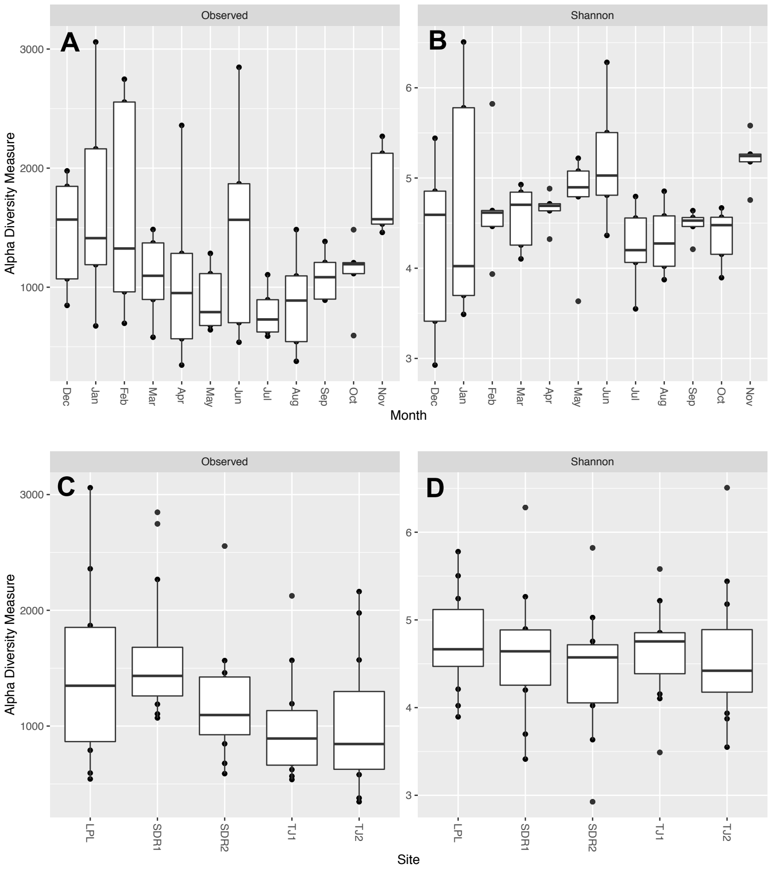

E

	Statistic (Kruskal-Wallis chi-squared)	*p-value*
Observed by Site	9.65	0.047
Observed by Month	17.14	0.104
Shannon by Site	1.73	0.785
Shannon by Month	16.58	0.121
