# Supplementary figures and images for: Pathogenic Vibrio Species Are Associated with Distinct Environmental Niches and Planktonic Taxa in Southern California (USA) Aquatic Microbiomes
Source: mSystems. 2021 Jul 6;6(4):e00571-21. doi: 10.1128/mSystems.00571-21 (PMC8407410; doi:10.1128/mSystems.00571-21)

**A**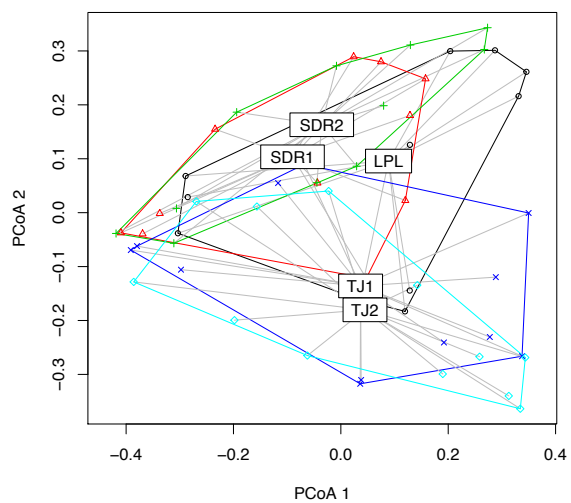**B**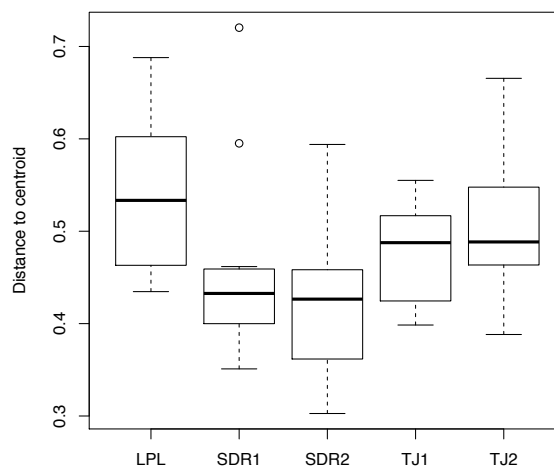**C**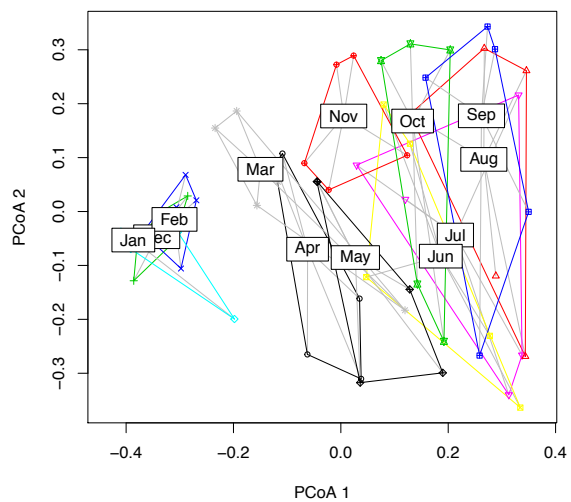**D**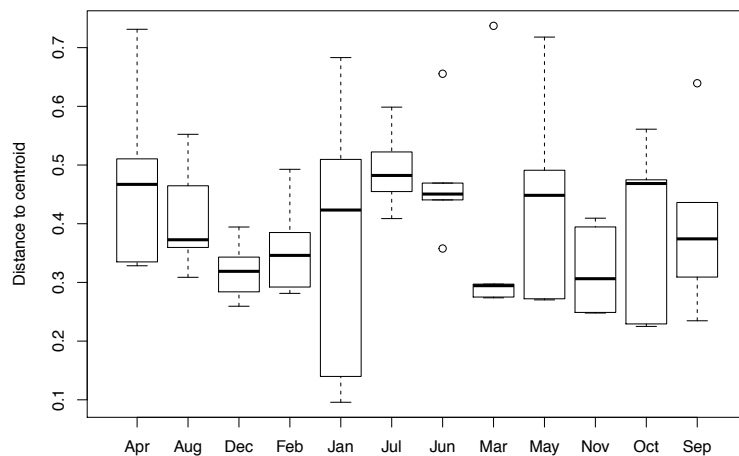

Supplement: FIG S4 [file msystems.00571-21-sf004.pdf]

**A**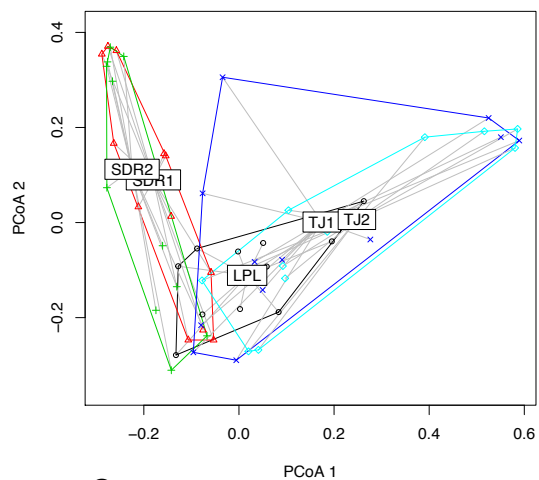**B**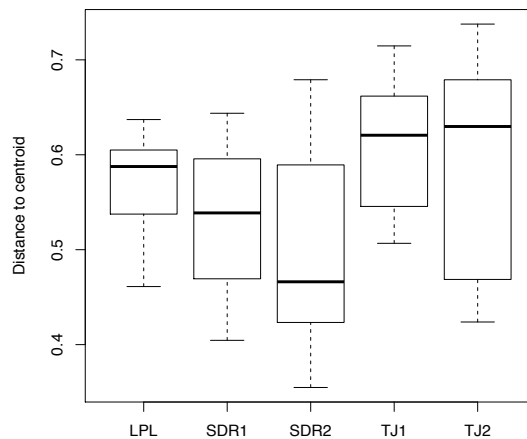**C**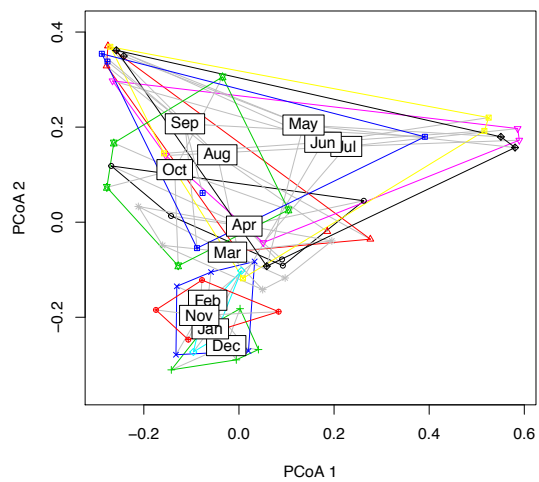**D**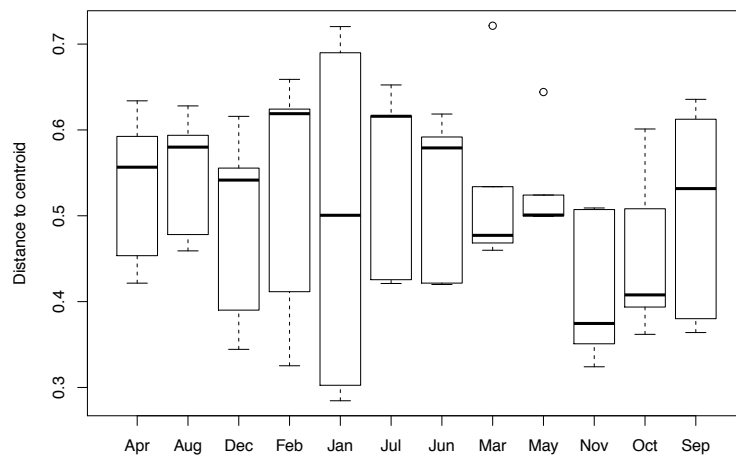

Supplement: FIG S5 [file msystems.00571-21-sf005.pdf]

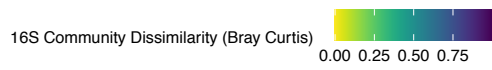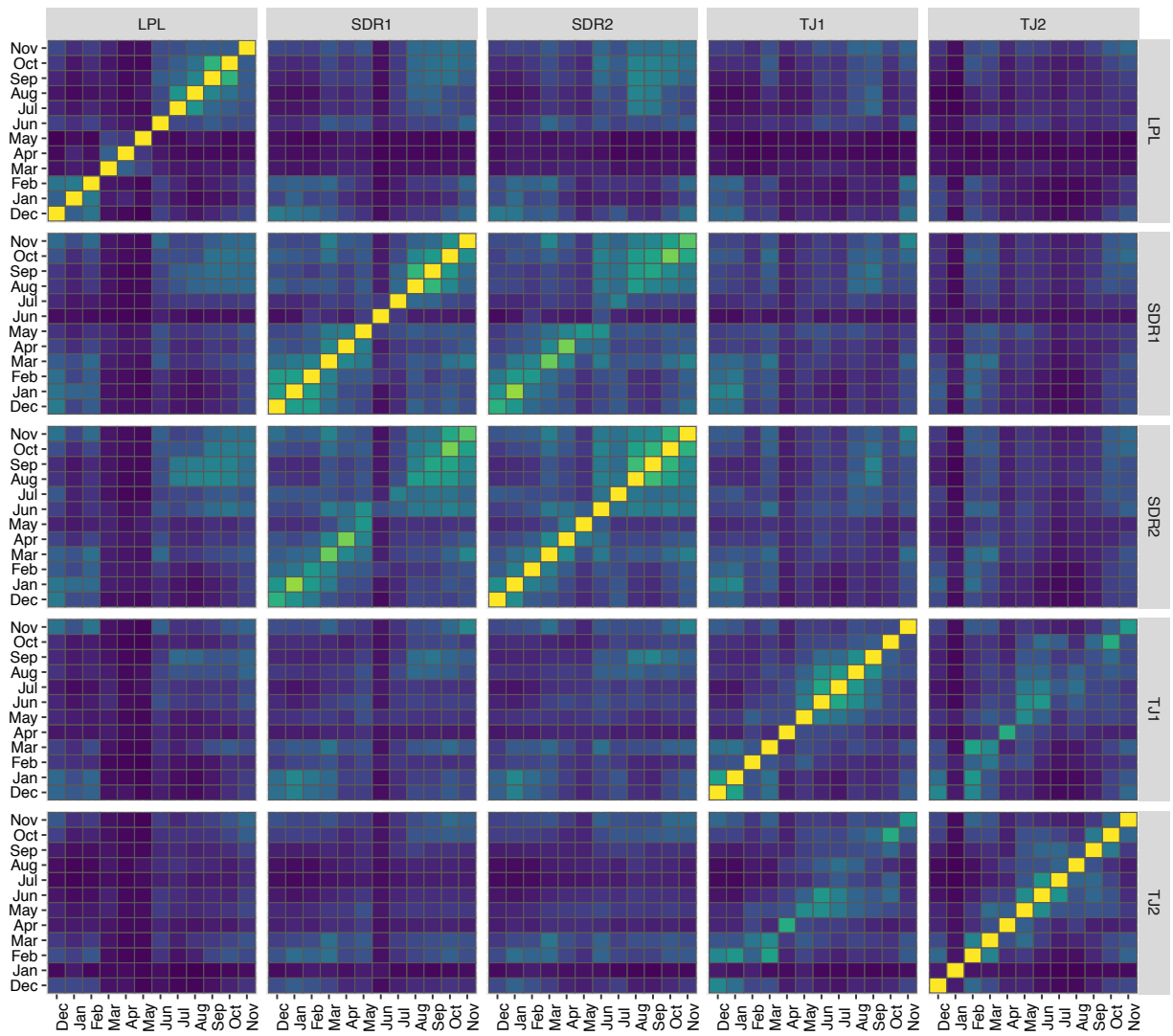

Supplement: FIG S6 [file msystems.00571-21-sf006.pdf]

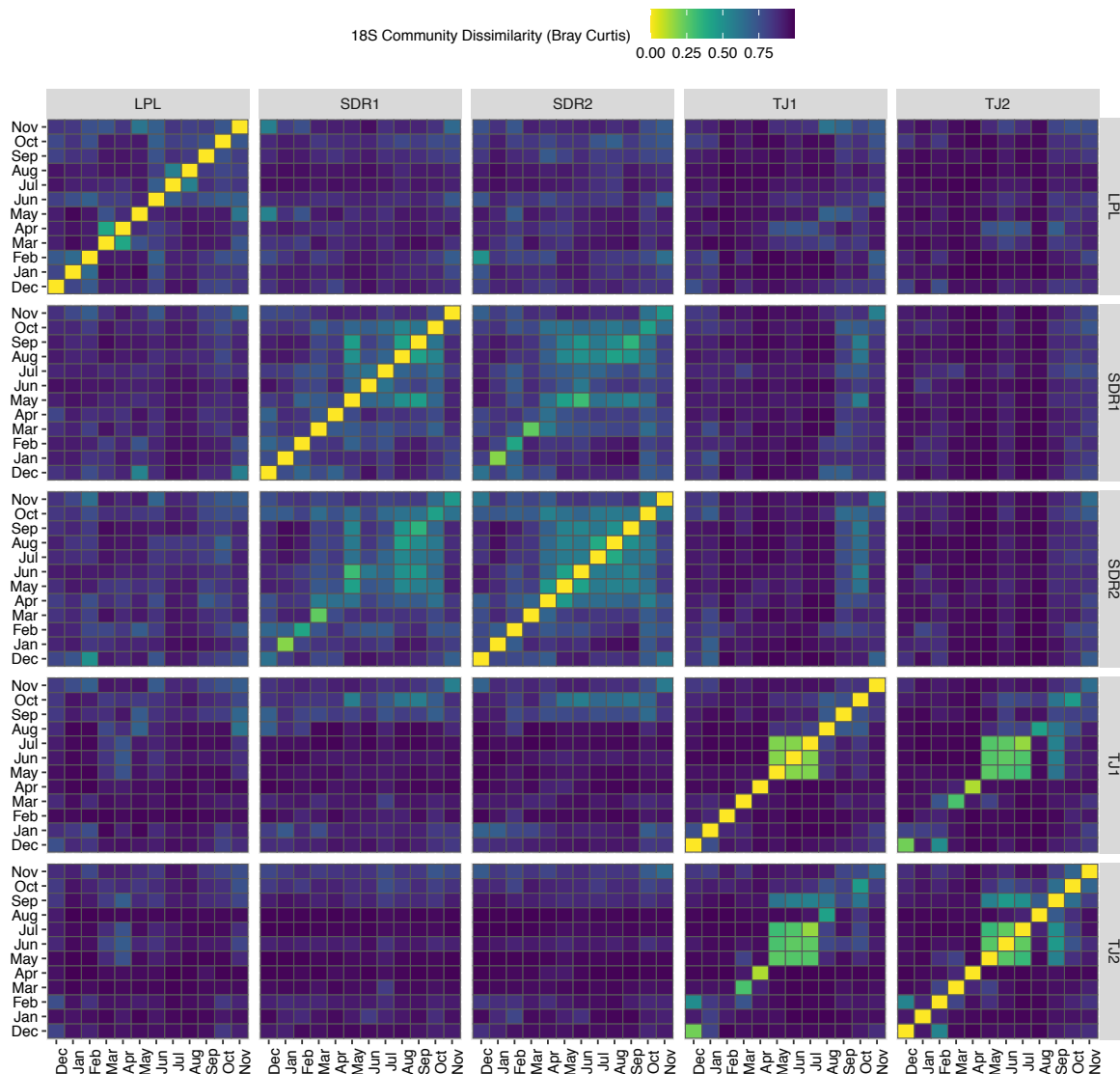

Supplement: FIG S7 [file msystems.00571-21-sf007.pdf]

**A**

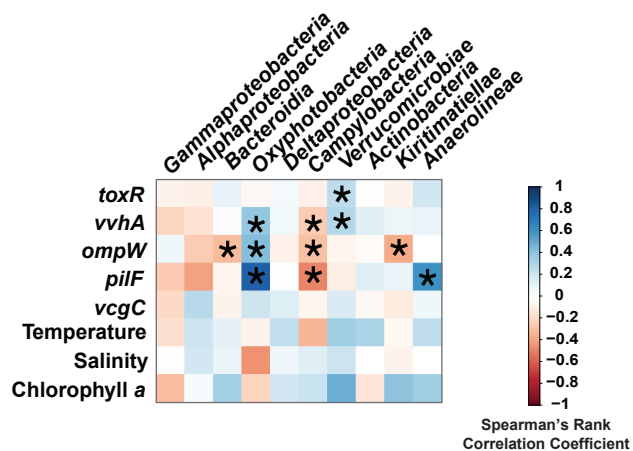

**B**

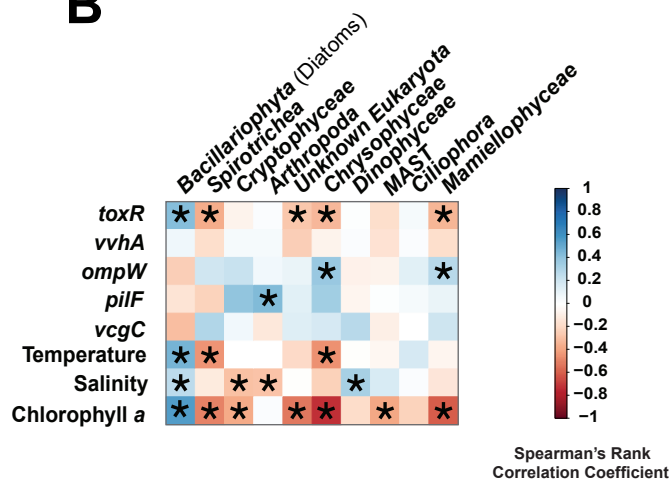

Supplement: FIG S8 [file msystems.00571-21-sf008.pdf]
