# Supplementary material for: Pathogenic Vibrio Species Are Associated with Distinct Environmental Niches and Planktonic Taxa in Southern California (USA) Aquatic Microbiomes
Source: mSystems. 2021 Jul 6;6(4):e00571-21. doi: 10.1128/mSystems.00571-21 (PMC8407410; doi:10.1128/mSystems.00571-21)
Supplement: TABLE S1 [file msystems.00571-21-st001.pdf]

Target Species	Target Gene	Primer Name	Sequence	Ta (ºC)	Study
V. vulnificus	*vcgC*	VVC-FW	AAAACTCATTGARCAGTAACGAAA	60	Baker-Austin et al. 2010
V. vulnificus	*vcgC*	VVC-REV	AGCTGGATCTAAKCCCAATGC	60	Baker-Austin et al. 2010
V. vulnificus	*vcgC*	VVC-Probe	/5HEX/AATTAAAGC/ZEN/CGTCAAGCC ACTTGACTGTAA/3IABkFQ/	60	Baker-Austin et al. 2010
V. vulnificus	*pilF*	PILF-FW	GATTGACTACGAYCCACACCG	60	Baker-Austin et al. 2012
V. vulnificus	*pilF*	PILF-REV	GRCGCGCTTGGGTGTAG	60	Baker-Austin et al. 2012
V. vulnificus	*pilF*	PILF-PROBE	/56-FAM/TGCTCAACC/ZEN/TCGCTAAGTT GGAAATCGATA/3IABkFQ/	60	Baker-Austin et al. 2012
V. parahaemolyticus	*toxR*	TOXR-FW	GAACCAGAAGCGCCAGTAGT	58	Taiwo et al. 2017
V. parahaemolyticus	*toxR*	TOXR-REV	AAACAAGCAGTACGCAAATCG	58	Taiwo et al. 2017
V. parahaemolyticus	*toxR*	TOXR-Probe	/5HEX/TCACAGCAG/ZEN/AAGCCACAG GTGC/3IABkFQ/	58	Taiwo et al. 2017
V. vulnificus	*vvhA*	VVHA-FW	TGTTTATGGTGAGAACGGTGACA	58	Campbell and Wright 2003
V. vulnificus	*vvhA*	VVHA- REV	TTCTTTATCTAGGCCCCAAACTTG	58	Campbell and Wright 2003
V. vulnificus	*vvhA*	VVHA-Probe	/56-FAM/CCGTTAACC/ZEN/GAACCACCCG CAA/3IABkFQ/	58	Campbell and Wright 2003
V. cholerae	*ompW*	ompW-F	TCAATGATAGCTGGTTCCTCAAC	58	Garrido-Maestu et al. 2014
V. cholerae	*ompW*	ompW-R	CGATGATAAATACCCAAGGATTGA	58	Garrido-Maestu et al. 2014
V. cholerae	*ompW*	ompW-Probe	/5HEX/TGGTATGCC/ZEN/AATATTGAA ACAACG/3IABkFQ/	58	Garrido-Maestu et al. 2014
