# Supplementary material for: Pathogenic Vibrio Species Are Associated with Distinct Environmental Niches and Planktonic Taxa in Southern California (USA) Aquatic Microbiomes
Source: mSystems. 2021 Jul 6;6(4):e00571-21. doi: 10.1128/mSystems.00571-21 (PMC8407410; doi:10.1128/mSystems.00571-21)
Supplement: TABLE S2 [file msystems.00571-21-st002.pdf]

Site	Month	*toxR* copies/ 100 mL: *V. parahaemolyticus* species	*vvhA* copies/ 100 mL: *V. vulnificus* species	*ompW* copies/ 100 mL: *V. cholerae* species	*pilF* copies/ 100 mL: *V. vulnificus* virulence	*vcgC* copies/ 100mL: *V. vulnificus* virulence	High abundance of one or more species (>1000 copies/ mL)	Isolate Shotgun Sequencing
LPL	December	0	0	12	NA	NA		
LPL	January	0	0	16	NA	NA		
LPL	February	0	0	0	NA	NA		Yes
LPL	March	476	0	31824	NA	NA	Yes	Yes
LPL	April	756	560	1151	1162	0	Yes	
LPL	May	788	1649	288864	74	168	Yes	Yes
LPL	June	1253	105	19	112	49	Yes	
LPL	July	53	0	0	NA	NA		Yes
LPL	August	133	42	8	0	0		Yes
LPL	September	152	16	67	0	0		
LPL	October	179	0	0	NA	NA		
LPL	November	215	0	0	NA	NA		
SDR1	December	203	0	NA	NA	NA		
SDR1	January	0	90	0	0	0		
SDR1	February	0	0	0	NA	NA		Yes
SDR1	March	641	0	29	NA	NA		Yes
SDR1	April	1124	3675	NA	1250	210	Yes	
SDR1	May	3045	13020	0	7350	0	Yes	Yes
SDR1	June	6510	1491	0	1155	0	Yes	
SDR1	July	2940	347	29	95	0	Yes	Yes
SDR1	August	399	0	0	NA	NA		Yes
SDR1	September	467	0	0	NA	NA		
SDR1	October	882	0	0	NA	NA		
SDR1	November	168	0	0	NA	NA		
SDR2	December	0	0	19	NA	NA		
SDR2	January	91	0	19	NA	NA		
SDR2	February	287	0	0	NA	NA		Yes
SDR2	March	756	0	0	0	868		Yes
SDR2	April	882	3780	29	872	0	Yes	
SDR2	May	1964	14175	286	7560	0	Yes	Yes
SDR2	June	189	63	0	0	0		
SDR2	July	959	0	19	NA	NA		Yes
SDR2	August	0	47	0	0	0		Yes
SDR2	September	53	0	0	NA	NA		
SDR2	October	205	0	0	NA	NA		
SDR2	November	142	0	0	NA	NA		
TJ1	December	32	0	50	NA	NA		
TJ1	January	0	0	0	NA	NA		
TJ1	February	1008	0	0	NA	NA	Yes	Yes

TJ1	March	195	180	0	0	0		Yes
TJ1	April	21630	126	0	0	0	Yes	
TJ1	May	284	0	0	0	0		Yes
TJ1	June	1071	0	0	NA	NA	Yes	
TJ1	July	420	0	0	NA	NA		
TJ1	August	704	0	0	NA	NA		Yes
TJ1	September	397	0	0	NA	NA		
TJ1	October	63	0	0	0	0		
TJ1	November	21	0	0	NA	NA		
TJ2	December	32	0	0	NA	NA		
TJ2	January	0	0	0	NA	NA		
TJ2	February	105	0	0	NA	NA		Yes
TJ2	March	126	98	0	0	0		Yes
TJ2	April	15015	95	0	0	0	Yes	
TJ2	May	6930	63	0	0	84	Yes	Yes
TJ2	June	3537	0	0	NA	NA	Yes	
TJ2	July	1218	0	0	NA	NA	Yes	
TJ2	August	788	0	0	NA	NA		Yes
TJ2	September	33530	0	16	0	0	Yes	
TJ2	October	6825	74	0	NA	NA	Yes	
TJ2	November	0	0	0	NA	NA		
